# Emerging evidence on Monkeypox: resurgence, global burden, molecular insights, genomics and possible management

**DOI:** 10.3389/fcimb.2023.1134712

**Published:** 2023-04-19

**Authors:** Ruchi Sharma, Kow-Tong Chen, Rohit Sharma

**Affiliations:** ^1^ Department of Rasa Shastra and Bhaishajya Kalpana, Faculty of Ayurveda, Institute of Medical Sciences, Banaras Hindu University (BHU), Varanasi, Uttar Pradesh, India; ^2^ Department of Occupational Medicine, Tainan Municipal Hospital (managed by Show Chwan Medical Care Corporation), Tainan, Taiwan; ^3^ Department of Public Health, College of Medicine, National Cheng Kung University, Tainan, Taiwan

**Keywords:** monkeypox, infection, genomics, molecular insights, vaccines, herbs, treatment

## Abstract

An outbreak of monkeypox (encoded enveloped double stranded DNA), resurgence and expansion has emerged in early 2022, posing a new threat to global health. Even though, many reports are available on monkeypox, still a comprehensive updated review is needed. Present updated review is focused to fill the research gaps pertaining to the monkeypox, and an extensive search was conducted in a number of databases, including Google Scholar, Scopus, Web of Science, and Science Direct. Although the disease usually progresses self-limiting, some patients require admission for kidney injury, pharyngitis, myocarditis, and soft tissue super infections. There is no well-known treatment available yet; still there has been a push for the use of antiviral therapy and tecovirimat as a promising option when dealing with co-morbidities. In this study, we mapped and discussed the updates and scientific developments surrounding monkeypox, including its potential molecular mechanisms, genomics, transmission, risk factors, diagnosis, prevention, vaccines, treatment, possible plant-based treatment along with their proposed mechanisms. Each day, a growing number of monkeypox cases are reported, and more cases are expected in the near future. As of now, monkeypox does not have a well-established and proven treatment, and several investigations are underway to find the best possible treatment from natural or synthetic drug sources. Multiple molecular mechanisms on pathophysiological cascades of monkeypox virus infection are discussed here along with updates on genomics, and possible preventive and therapeutic strategies.

## 1 Introduction

An evolving zoonotic disease, monkeypox occurs due to a virus belonging to poxviridae family and orthopoxvirus genus (Lai et al., 2022). Along with cowpox virus, variola virus, and vaccinia virus, the monkeypox virus is one of four orthopox virus species that causes disease in humans (Shchelkunov et al., 2005). A monkeypox infection causes few symptoms identical to those of smallpox, but is less severe, and rarely causes death compared to that of smallpox (Petersen et al., 2019). Two outbreaks of a disease that appeared like pox, led to the discovery of monkeypox in 1958 which spread across colonies of monkeys (Mileto et al., 2022). Monkeypox may have been named so, due to its first-time appearance in monkeys and pox like symptoms, but its origin remains mysterious (Begum et al., 2023). The virus can, however, also be carried by African rodents and nonhuman primates (such as monkeys) and infect humans (Begum et al., 2023). Later an instance of monkeypox in a human was first reported in 1970 (Sejvar et al., 2004). By the time, number of countries in Central and Western Africa had already reported the monkeypox cases before the outbreak of 2022 (Simões and Bhagani, 2022). Prior to recently, nearly all monkeypox cases aside from Africa were associated with traveling overseas countries or importing animals that frequently suffer from the disease (Al-Tawfiq et al., 2022). It has been determined that the monkeypox outbreak has resulted in a public health emergency of International concern in multiple countries (Formenty et al., 2010).

Several mammalian species are known to be susceptible to monkeypox, however the native host of the disease is unidentified (Khodakevich et al., 1986). From times monkeypox virus isolates have known of two wild animal origins: a rope squirrel and a sooty mangabey from Ivory Coast and the Democratic Republic of Congo (DRC) places respectively (Khodakevich et al., 1986; Jezek et al., 1988; Hutson et al., 2009; Radonić et al., 2014). Monkeypox has a similar clinical picture to smallpox, but the major difference is the swelling of lymph nodes that begins early, often during fever (Sklenovská and Van Ranst, 2018) and has a lower mortality rate (Centers for Disease Control and Prevention (CDC), 2022). There can be a period of infection of up to four weeks before the lesion of monkeypox desquamates (Fenner et al., 1998). It is likely that patients will experience complications such as second level bacterial infections, bronchopneumonia, respiratory distress, dehydration, gastrointestinal complications, encephalitis, sepsis, and infection of cornea with blindness as a result (Elsevier – clinical overviews, 2022). At present, no specific treatment exists for monkeypox virus infections, instead patients being treated with symptomatic treatment and supportive care (Otu et al., 2022).

Even though many short studies are present, there is a need of study which covers the detailed explanation of the disease along with mechanism involved in it. So, this study was conducted in order to map and summarize upcoming scientific developments surrounding monkeypox, such as its potential molecular insights, genomic patterns, diagnosis, prevention, vaccines, treatment and possible plant-based leads of monkeypox. Presented below is a comprehensive updated review that illustrates a better understanding on monkeypox virus. Understanding the mechanisms involved in monkeypox infection will strengthen the disease preventive and therapeutic strategies along with the discovery of new drugs.

## 2 Methodology

**Figure 1:**
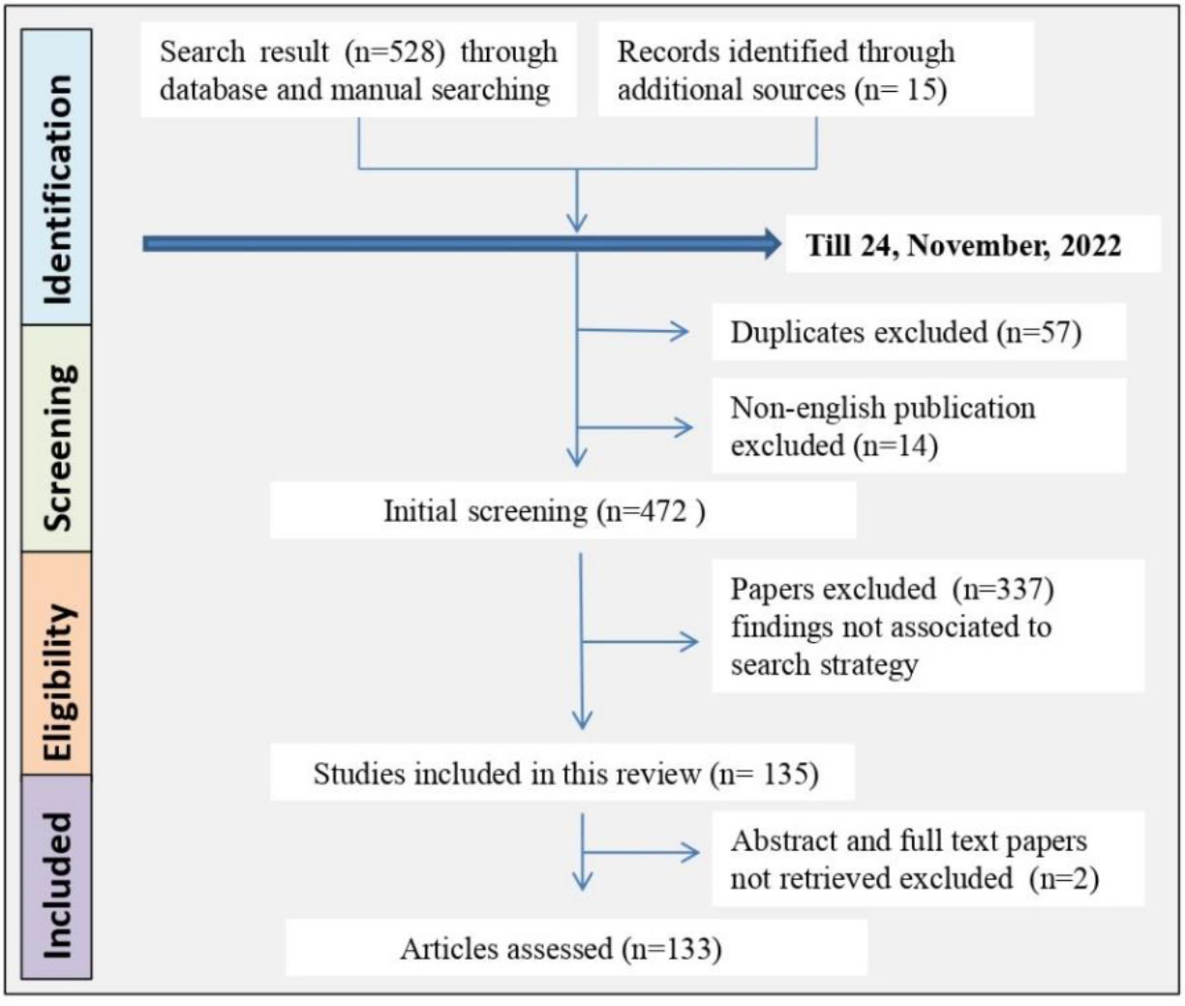
Flowchart showing screening methodology for the review.

We conducted a review based on scientific articles that addressed monkeypox and its diagnostic method or treatment, especially pharmacological properties (**Figure 1**). Science Direct, SciFinder, Google Scholar, MEDLINE, EMBASE, and Scopus were the search engines used to search for published articles (till 24, November, 2022). For extracting information about the effects of monkeypox on global transmission and treatment, we used keywords such as “monkeypox”, “transmission”, “mode of spread”, “fatality”, “incidence rate”, “prevention”, “diagnosis”, “vaccine”, “genomics”, “molecular mechanism”, “treatment”, “clinical trial”, “case study”, “traditional medicine”, and “mechanism of action”, along with their corresponding MeSH terms and conjunctions OR/AND. To understand the severity of disease and possible treatment options, we searched the available reports for scientific claims. All searches were limited to English. We excluded conference proceedings, gray literature, unpublished data, newspaper articles, preliminary reports without substantial proof of the claim, abstracts and full texts that could not be retrieved, and studies not relevant to this review. The graphs were generated using the GraphPad software version 6 for analysis.

## 3 Timeline recapped

**Figure 2:**
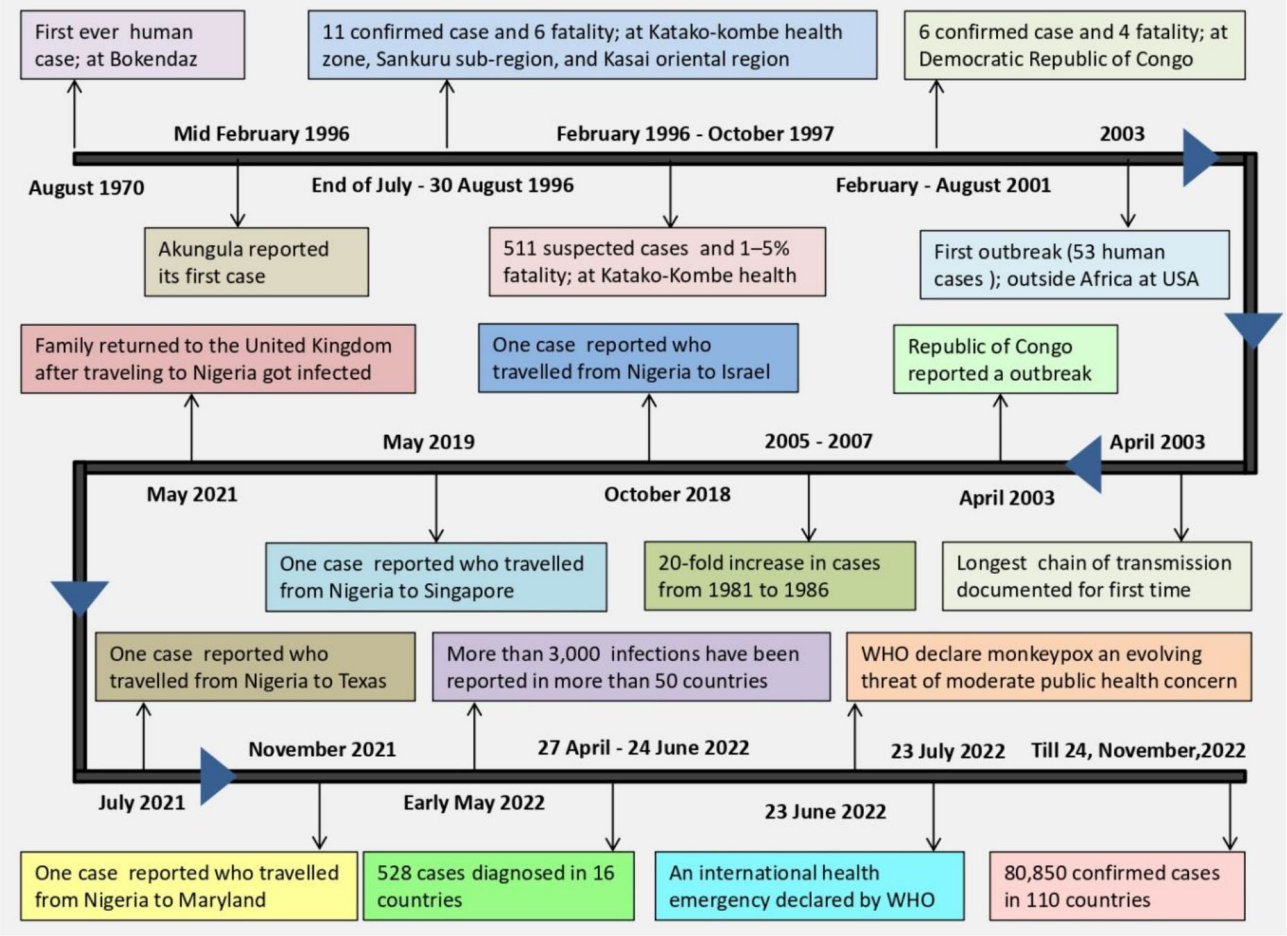
Timeline of events in monkeypox’s global transmission.

A non-fatal pox outbreak was first reported in 1958 by Von Magnus in Copenhagen in Cynomolgus macaque (C. macaque). Prior to 1970, no human infection with monkeypox virus had been documented (Breman et al., 1980; Sejvar et al., 2004). The following cases of human monkeypox have been reported worldwide since 1970 (**Figure 2**, **Table 1**) (World Health Organization, 1996; Merouze and Lesoin, 1983; World Health Organization, 2022; McConnell et al., 1962; Ladnyj et al., 1972; Breman et al., 1980; Massung et al., 1994; Centers for Disease Control and Prevention (CDC), 1997; Mukinda et al., 1997; Anderson et al., 2003; Centers for Disease Control and Prevention (CDC), 2003a; Centers for Disease Control and Prevention (CDC), 2003b; Huhn et al., 2005; Learned et al., 2005; Likos et al., 2005; Nalca et al., 2005; Reynolds et al., 2006; Hutson et al., 2007; Rimoin et al., 2010; Kantele et al., 2016; Kozlov, 2018; Beer and Bhargavi Rao, 2019; Bhattacharya et al., 2022; Luo and Han, 2022; Tomori and Ogoina, 2022). On June 23, 2022, the WHO declared monkeypox as an “evolving threat of moderate public health concern” (Reed et al., 2004). In a WHO report published on 23 July 2022, there were more than 16,000 cases with five deaths reported from 75 countries and territories (World Health Organisation, 2022). The outbreak of monkeypox in 2022 gained global attention when WHO declared it an “International Health Emergency” on July 27, 2022 (As monkeypox surges, WHO urges reducing number of sexual partners, 2022). Recently, in nearly 110 countries, 80,850 confirmed cases (79,877 in areas where monkeypox has not been reported historically and 973 in areas where monkeypox has been reported historically) and 55 deaths (42 in areas where monkeypox have not reported historically and 13 in areas where monkeypox have been reported historically) were reported till 24, November, 2022 (Monkeypox data explorer - our world in data, 2022).

**Table 1:** An overview of monkeypox outbreaks cases between August, 1970 and 24, November, 2022.

Year	1970–1979	1980–1989	1990–1999	2000–2009	2010–2019	2020–2021	24 May, 2022	Till 24, November, 2022
Country								
**Andorra**	–	–	–	–	–	–	–	4
**Argentina**	–	–	–	–	–	–	–	894
**Aruba**	–	–	–	–	–	–	–	3
**Australia**	–	–	–	–	–	–	2	141
**Austria**	–	–	–	–	–	–	1	326
**Bahamas**	–	–	–	–	–	–	–	2
**Bahrain**	–	–	–	–	–	–	–	1
**Barbados**	–	–	–	–	–	–	–	1
**Belgium**	–	–	–	–	–	–	4	789
**Benin**	–	–	–	–	–	–	–	3
**Bermuda**	–	–	–	–	–	–	–	1
**Bolivia**	–	–	–	–	–	–	–	252
**Bosnia and Herzegovina**	–	–	–	–	–	–	–	9
**Brazil**	–	–	–	–	–	–	–	**9876**
**Bulgaria**	–	–	–	–	–	–	–	6
**Cameroona**	1	1	–	–	16	–	–	16
**Canada**	–	–	–	–	–	–	15	**1449**
**Central African Republic**	–	8	–	–	61	–	2	12
**Chile**	–	–	–	–	–	–	–	**1259**
**China**	–	–	–	–	–	–	–	1
**Colombia**	–	–	–	–	–	–	–	**3803**
**Costa Rica**	–	–	–	–	–	–	–	23
**Croatia**	–	–	–	–	–	–	–	29
**Cuba**	–	–	–	–	–	–	–	8
**Curaçao**	–	–	–	–	–	–	–	3
**Cyprus**	–	–	–	–	–	–	–	5
**Czechia**	–	–	–	–	–	–	1	70
**DRC**	38	343	511	10,027	18,788	7,374	10	206
**Denmark**	–	–	–	–	–	–	2	191
**Dominican Republic**	–	–	–	–	–	–	–	52
**Ecuador**	–	–	–	–	–	–	–	346
**Egypt**	–	–	–	–	–	–	–	1
**El Salvador**	–	–	–	–	–	–	–	21
**Estonia**	–	–	–	–	–	–	–	11
**Finland**	–	–	–	–	–	–	–	42
**France**	–	–	–	–	–	–	5	**4104**
**Georgia**	–	–	–	–	–	–	–	2
**Germany**	–	–	–	–	–	–	12	**3672**
**Ghana**	–	–	–	–	–	–	–	107
**Gibraltar**	–	–	–	–	–	–	–	6
**Greece**	–	–	–	–	–	–	–	85
**Greenland**	–	–	–	–	–	–	–	2
**Guadeloupe**	–	–	–	–	–	–	–	1
**Guatemala**	–	–	–	–	–	–	–	141
**Guyana**	–	–	–	–	–	–	–	2
**Honduras**	–	–	–	–	–	–	–	11
**Hong Kong**	–	–	–	–	–	–	–	1
**Hungary**	–	–	–	–	–	–	–	80
**Iceland**	–	–	–	–	–	–	–	16
**India**	–	–	–	–	–	–	–	17
**Indonesia**	–	–	–	–	–	–	–	1
**Iran**	–	–	–	–	–	–	–	1
**Ireland**	–	–	–	–	–	–	–	217
**Israel**	–	–	–	–	1	–	1	262
**Italy**	–	–	–	–	–	–	5	917
**Jamaica**	–	–	–	–	–	–	–	16
**Japan**	–	–	–	–	–	–	–	7
**Jordan**	–	–	–	–	–	–	–	1
**Latvia**	–	–	–	–	–	–	–	6
**Lebanon**	–	–	–	–	–	–	–	18
**Liberia**	4	–	–	–	6		–	3
**Lithuania**	–	–	–	–	–	–	–	5
**Luxembourg**	–	–	–	–	–	–	–	57
**Malta**	–	–	–	–	–	–	–	33
**Martinique**	–	–	–	–	–	–	–	1
**Mexico**	–	–	–	–	–	–	–	**3145**
**Moldova**	–	–	–	–	–	–	–	2
**Monaco**	–	–	–	–	–	–	–	3
**Montenegro**	–	–	–	–	–	–	–	2
**Morocco**	–	–	–	–	–	–	–	3
**Mozambique**	–	–	–	–	–	–	–	1
**Netherlands**	–	–	–	–	–	–	6	**1248**
**New Caledonia**	–	–	–	–	–	–	–	1
**New Zealand**	–	–	–	–	–	–	–	35
**Nigeria**	4	–	–	–	228	42	–	624
**Norway**	–	–	–	–	–	–	–	93
**Panama**	–	–	–	–	–	–	–	40
**Paraguay**	–	–	–	–	–	–	–	17
**Peru**	–	–	–	–	–	–	–	**3444**
**Philippines**	–	–	–	–	–	–	–	4
**Poland**	–	–	–	–	–	–	–	213
**Portugal**	–	–	–	–	–	–	39	948
**Qatar**	–	–	–	–	–	–	–	5
**Republic of the Congo**	–	–	–	73	24	–	–	5
**Romania**	–	–	–	–	–	–	–	45
**Russia**	–	–	–	–	–	–	–	2
**Saint Martin**	–	–	–	–	–	–	–	1
**San Marino**	–	–	–	–	–	–	–	1
**Saudi Arabia**	–	–	–	–	–	–	–	8
**Serbia**	–	–	–	–	–	–	–	40
**Singapore**	–	–	–	–	1	–	–	19
**Slovakia**	–	–	–	–	–	–	–	14
**Slovenia**	–	–	–	–	–	–	1	47
**South Africa**	–	–	–	–	–	–	–	5
**South Korea**	–	–	–	–	–	–	–	3
**South Sudan**	–	–	–	–	19	–	–	18
**Spain**	–	–	–	–	–	–	45	**7405**
**Srilanka**	–	–	–	–	–	–	–	1
**Sweden**	–	–	–	–	–	–	1	220
**Switzerland**	–	–	–	–	–	–	2	546
**Taiwan**	–	–	–	–	–	–	–	4
**Thailand**	–	–	–	–	–	–	–	12
**Turkey**	–	–	–	–	–	–	–	12
**Ukraine**								5
**United Arab Emirates**	–	–	–	–	–	–	1	16
**United Kingdom**	–	–	–	–	4	–	71	**3720**
**United States**	–	–	–	47	–	2	4	**29199**
**Uruguay**	–	–	–	–	–	–	–	14
**Venezuela**	–	–	–	–	–	–	–	10
**Vietnam**	–	–	–	–	–	–	–	2
**Total cases (till 24, November,2022)**								80,850

*Countries with more than 1000 cases are bold.

## 4 Monkeypox virus strains: Genomic perspective

**Figure 3:**
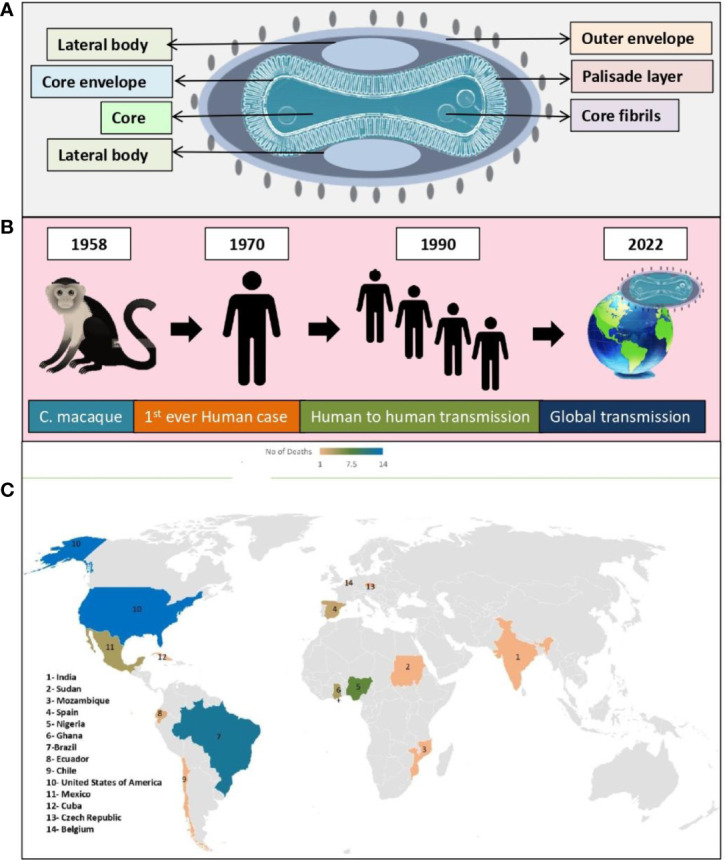
Monkeypox virus; **(A)** Structure, **(B)** Global transmission, **(C)** Reported death cases of different geographical areas (till 24, November, 2022).

**Figure 3A** shows structure of the double stranded DNA virus (monkeypox virus), which causes monkeypox to occur in humans and animals (**Figure 3B**). This virus belonged to the poxviridae family and referred to as orthopoxviruses. As with variola, cowpox, and vaccinia, monkeypox virus belongs to the orthopoxvirus genus. It is not related to, nor is it descended from, the variola virus, which is responsible for smallpox (von Magnus et al., 1959; Breman et al., 1980; Shchelkunov et al., 2002; Alkhalil et al., 2010).

Two genetic clades have been identified in monkeypox virus, namely Central African clade (recently renamed as Clade I) and West African clade (recently renamed as Clade II). Furthermore, Clade II has two subclades: IIa and IIb, referring to the set of mutations that are circulating in the 2022 worldwide pandemic. There is a geographic separation between these two clades, as well as a distinction between their epidemiological and clinical characteristics. It has never been documented that West African clade transmits from case to case, and there is a case fatality rate (CFR) of 1%. As a contrast, the Congo Basin clade (aka Central African clade) exhibits a CFR of 11%, and transmission has been documented up to six times sequentially. It is believed that isolates from West African clade came from Nigeria, Ivory Coast, Liberia, USA, and Sierra Leone (imported from Ghana). On the other hand, isolates from Central African clade come from Congo, Gabon, Central African Republic, Cameroon, Sudan, and DRC. Non-vaccinated individuals of Central African encountered a 10% death rate in the 1980s, while cases occurring in West African strains were not fatal. It has been confirmed that the 2022 monkeypox virus is of West African origin based on preliminary genetic data. Various studies were conducted to compare genomes and was found that nucleotide sequences of the Central African strain (ZAI-96) differ by 0.55-0.56% from those of 3 West African strains (SL-V70, WRAIR-61 and COP-58). Central and West African strains have 173 and 171 unique functional genes, respectively. In terms of protein sequence, they have about 99.4% identity and share 170 orthologs. Transcription regulatory sequences between the two genomes did not differ significantly. In order to determine which virulence genes are present in both strains, researchers studied 56 virulence genes to determine which are present in both strains, 53 of which were found common to both. 276 substitutions have been found in these 53 genes, accounting for 93 non-conservative changes, 61 conservative changes, and 121 silent changes in amino acid. In total, 16 proteins have extended their N- and C-termini, altering their predicted size. A study reported that BR-203, COP-C3L, and BR-209 orthologs accounted for significant virulence differences between the two strains, which is supported by another study reporting similar gene candidates (Jezek and Fenner, 1988; von Magnus et al., 1959; Ladnyj et al., 1972; Ježek et al., 1987; Chen et al., 2005; Likos et al., 2005; Osorio and Yuill, 2008; Weaver and Isaacs, 2008; Monkeypox: experts give virus variants new names, 2022; Comparative pathology of north American and central African strains of monkeypox virus in a ground squirrel model of the disease, 2022; Multi-country monkeypox outbreak: situation update, 2022).

Monkeypox virus encoded of all the orthopoxvirus genes known so far, but have only small subsets of the genes related to host range and immunomodulation (Shchelkunov et al., 2002). Monkeypox may adapt further to become a more effective human pathogen by a series of extended person-to-person transmissions. Moreover, as the human population changes, ecological disturbances occurs and new infections emerges such as the HIV and COVID-19, which can be a possible cause of decreased immunity and further contributing towards more spreading of monkeypox virus (Chen et al., 2005; Likos et al., 2005; Weaver and Isaacs, 2008).

Also, an analysis of publicly available genomes from the current human monkeypox virus outbreak of 2022 was conducted to determine the phylogenetic diversity. As compared to previous monkeypox virus outbreaks, this outbreak reveals a distinct monophyletic lineage. Virus outbreaks in Europe may have started as early as March 2022, according to studies. As compared to its related viral predecessors from 2018 and 2019, monkeypox virus 2022 exhibits a marked divergence. In contrast to what is normally observed in orthopoxviruses, the heightened mutational signature this time indicates an accelerated evolutionary path. There has been an increase in cases reported across multiple regions, suggesting that some of these changes in genomics have led to more efficient transmission and dispersal mechanisms, which are compatible with sexual transmission, but research is needed to confirm this assertion. In addition, host-specific mechanisms, like APOBEC enzymatic editing, may be driving this rapid evolution of virus in favor of emergence of a host-specific clade that has enhanced human-to-human transmission capabilities. In light of its continuous spread across different countries and the increasing number of cases reported globally, genomic variability is likely to increase continuously its transmission efficiency (Kraemer et al., 2022; Luna et al., 2022).

In another study on monkeypox virus genome comparisons, indicate that COP-C3L ortholog, a gene that codes for a complement control protein associated to innate immune response, may play a role in determining the degree of virulence among those strains. One SCR domain is truncated in the Central African ortholog of COP-C3L, which is likely to affect its function, particularly its decay-accelerating ability. The monkeypox strain of Central Africa also contains truncated COP-E3L and COP-K3L, which is similar to the proteins in variola and vaccinia and allows the organism to resist interferons (IFN). Unlike COP-E3L and COP-K3L, monkeypox virus has a full-length ortholog of BR-203 (protein that prevents lymphocytes apoptosis), but variola virus lacks it and vaccinia virus has fragments. The differences between these three orthopoxviruses in terms of virulence may be explained by their proteins differences which affect the host immune system. Research is needed to determine whether genes encoding of proteins fragments with known functions influence its virulence. It may be possible to develop safer vaccines and better therapeutics with such knowledge (Carter et al., 2005; Reynolds et al., 2017).

## 5 Molecular perspective

As molecular biology has advanced, we have gained a deeper understanding of how viruses replicate and infect cells. The genome of this organism is relatively larger containing approximately 1,96,858 number of base pairs, which encodes for 190 open reading frames (Shchelkunov et al., 2002). These open reading frames of virus make up most of the replication material in the infected cell. Viral entry into cells depends on the cell type and viral strain and is initiated once the virus attaches to the cell surface through the interaction between multiple viral ligands and cell receptors (Carter et al., 2005).

An apoptosis is a natural mechanism that occurs when a variety of stimuli trigger the death of cells for maintaining homeostasis in tissue and removal of abnormal or infected cells (Hengartner, 2000). Due to the importance of apoptosis in the immune response, poxviruses developed several anti-apoptotic strategies to disrupt it (Everett and McFadden, 2002). In order to survive, viruses contain many proteins that interfere with apoptosis at various points (Elmore, 2007). Previous study data however indicate that NOXA and caspase-3 were upregulated, while Bcl-2, PUMA, and PAK2 were downregulated in monkeypox virus, which is consistent with apoptosis induction (Alkhalil et al., 2010). Apoptosis-specific genes are underregulated in cells infected with monkeypox virus, whereas anti-apoptotic outcomes are observed in other poxvirus infected cells, suggesting that monkeypox virus infects cells in a way that is anti-apoptotic *via* a mechanism downstream from apoptosis initiation (Nichols et al., 2017). Although monkeypox virus genes involved in blocking apoptosis are unknown, still data suggest that an orthologs of the vaccinia virus (F1L) gene functions in the same manner as Bcl-2 by directly targeting mitochondria (Alkhalil et al., 2010).

Also, studies suggest that expression of histone, modification in histone posttranslational, and chromatin dynamic exchanges play an important role in host-poxvirus interactions (Alkhalil et al., 2010; Nichols et al., 2017). Moreover, studies introduced signaling components that regulate actin cytoskeletal dynamics as infection-regulated genes which further helps in regulating microtubule signaling. A membrane-associated protein encoded by the intersection 1 gene (SH3 domain protein) is said to be closely related to actin assembly machinery that controls endocytic membrane traffic. Morphological differentiation and cell motility are also affected by Rho-effector ROCK1, which is also closely related to cytoskeletal dynamics. As well as RAS p21 protein activator, homolog of oncogene from v-Ki-rat sarcoma virus and SOS2 are found essential in polymerizing actin filaments and reorganizing the cytoskeleton (McGlade et al., 1993; Giancotti and Ruoslahti, 1999; Pawlak and Helfman, 2002; Scaife et al., 2003; Alkhalil et al., 2010).

Studies also suggest that genes related to ion channels were impacted by monkeypox virus infection in an intriguing and novel manner. A total of ten genes that encode nine ion channels and a transporter were suppressed as infection progressed. A large number of these channels are located on the membrane of the cell in order to maintain its osmolarity homeostasis and cell membrane potential. Modulation mechanisms of transport, such as indirect consequences of Ras, Rho, and Rab GTPases, have been described, but their impact on viral infections and global cell biology continues to be unclear. However, there has been a report found describing how myxoma poxvirus protein M11L hinders apoptosis by interacting with mitochondrial permeability transition pores (Everett et al., 2002; Pochynyuk et al., 2007).

## 6 Monkeypox: A serious global health threat?

The monkeypox virus, is a public health concern since it can be transmitted from infected individuals, animals and contaminated substances. A number of countries that are non-endemic were reported to have monkeypox in May 2022 (Bunge et al., 2022). According to our discussion above, monkeypox lethality varies across Africa, suggesting its growing threat. Also, the current outbreak of monkeypox virus infection in humans suggests that biological aspects of virus, human behavior or both, have changed (Quarleri et al., 2022). A number of factors may have contributed to these changes, including the decline in smallpox immunity, the relaxed prevention measures for COVID -19, the resumption of international travel, the change in sexual interaction, and the presence of large gatherings in large numbers (Vaughan et al., 2018; Monkeypox update from AR Dept. of health - Arkansas medical society, 2022; Sharma V. et al., 2022; Thornhill et al., 2022). In the course of time, all viruses change and evolve despite this, monkeypox viruses mutate more slowly than COVID-19 virus (León-Figueroa et al., 2022). Currently, many studies are being conducted to understand the epidemiology, sources, and patterns of infection (Centers for Disease Control and Prevention, 2022; Ligon, 2004; Erez et al., 2019; Yong et al., 2020). Out of which study conducted in African countries by the European Centre for Disease Prevention and Control estimates that immunocompromised people, children, and young adults are at higher risk of death (Dhawan et al., 2022). This outbreak reported total of 55 deaths until know among various geographical areas (**Figure 3C**).

Policy makers and government officials also need to understand public opinion regarding the health crisis in order to develop health policies for monitoring and controlling it. So, in order to determine public attitudes towards the monkeypox virus, an analysis was conducted on 27, June, 2022 using techniques of advanced machine learning, particularly technique of Natural Language Processing (NLP). Intriguingly, the analysis results indicate that more people are posting positively about the monkeypox virus on social media (28.82%) than posting negatively about it (23.11%). When examining the tweets more closely, most positive tweets about monkeypox was that this virus is not severe and there is a low death rate caused by the disease. There are only few tweets about monkeypox that have negative sentiments suggesting that public hasn’t panicked that much about the virus, and that too negative comments are of people discussing death caused by monkeypox, virus severity, infections caused due to it, whether it can be transmitted, vaccines available, whether it will be the next pandemic after COVID-19, if it is safe to travel, if it will affect the schools functioning, and whether it will affect the people lives. In light of the early stages of this epidemic, researchers and policymakers can use this study to better understand public concerns about monkeypox and develop effective awareness programs and control the outbreak so that general public concerns can be addressed. In order to raise awareness about the ongoing outbreak, it is imperative to teach the general public what the disease symptoms are and when to seek medical attention (Farahat et al., 2022; Sv and Ittamalla, 2022).

## 7 Transmission

Over the past several years, numerous outbreaks of emerging infectious viral diseases originating from zoonotic animals including MERS-CoV, SARS-CoV-2, H7N9 (avian influenza), chikungunya virus, ebola virus, dengue virus, and Japanese encephalitis virus, which all are highly pathogenic have been reported. From time to time, international travelers reported to spread these viruses. SARS-CoV-2 was spread across countries largely due to international travel. In some non-African countries, monkeypox outbreaks have been reported recently. As of now, there are no links between monkeypox endemic areas and travel found in the current scenario. It is imperative to investigate the root cause of the current outbreaks as soon as possible. Furthermore, the focus must be placed on determining the zoonotic reservoir, zoonosis, spill overs from the host, etc., of this virus. Despite a lack of association between monkeypox outbreaks and air travel, it might be more likely that the disease will spread as a result of air travel as it is a contagious disease (Morens and Fauci, 2020; Wells et al., 2020; Bhattacharya et al., 2022; Bryer et al., 2022; Sharma R. et al., 2022; Begum et al., 2023; Varghese et al., 2023a).

**Figure 4:**
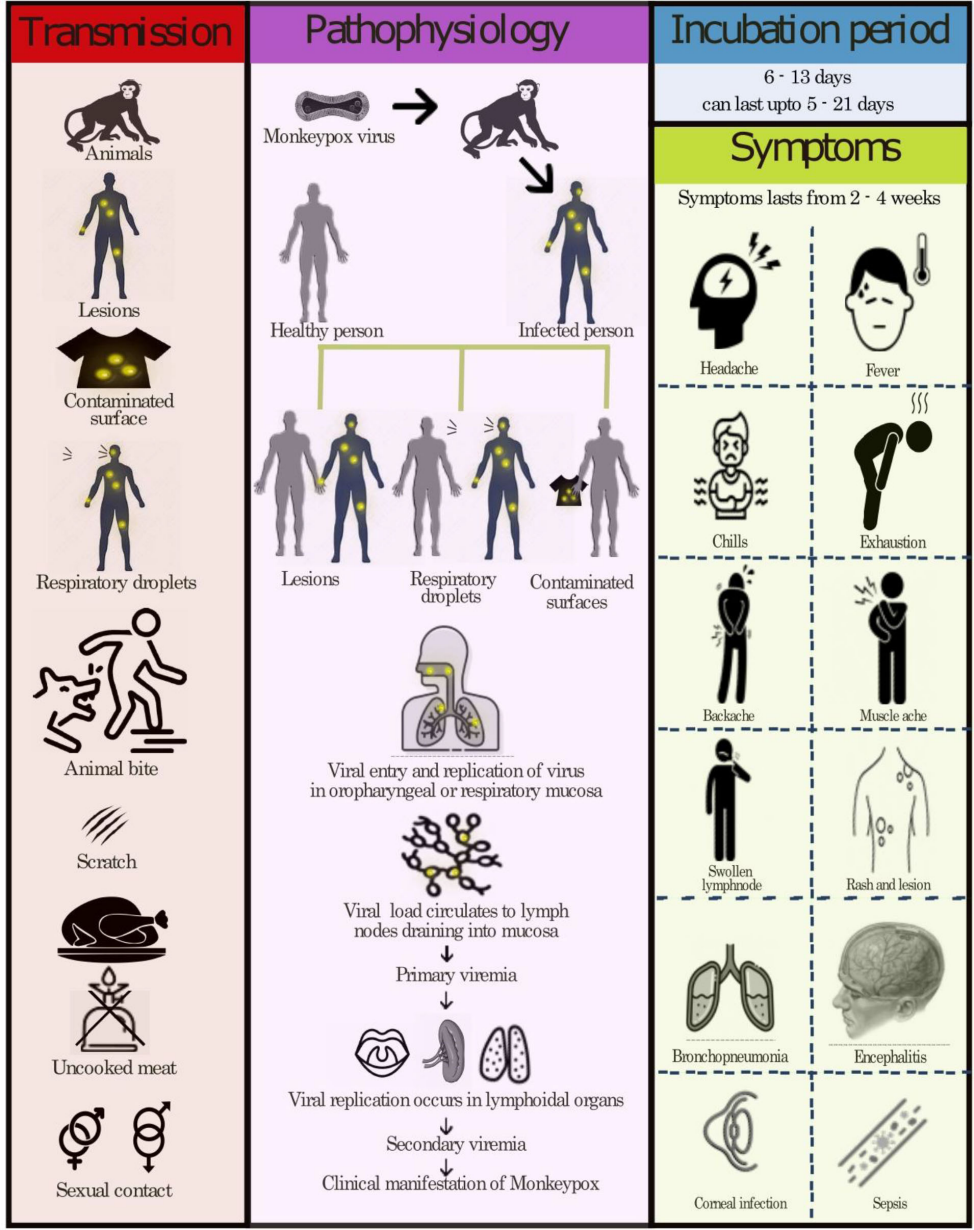
Transmission, pathophysiology and symptoms of monkeypox.

A person who suffers from monkeypox can transmit it to others from the time period of beginning of symptoms until the complete healing of rash and formation of fresh skin layer (Farahat et al., 2022; Soriano and Corral, 2022; Vivancos et al., 2022; Zambrano et al., 2022). The possible causes for anyone to contract monkeypox up to its clinical manifestation are described in **Figure 4**. In addition to it, placental transmission can also spread the virus to a fetus while a mother is pregnant. Current spread has disproportionately affected gay, men who have sexual relations with men and bisexual men by developing a vesicular pustular rash or lesions on genital, suggesting that sexual networks are amplifying transmission and close contact is believed to constitute a reason of disease transmission (Farahat et al., 2022). This rash typically occurs on the perennial area and genitals, suggesting that it was transmitted sexually, but it can be mistaken for molluscum contagiosum, chancre, herpes simplex infection or granuloma inguinal (Farahat et al., 2022; Soriano and Corral, 2022; Vivancos et al., 2022; Zambrano et al., 2022).

Moreover, infected human fecal sample can also contain monkeypox virus DNA, according to a recent small study, which may provide yet another potential route for viral transmission. Since some animals (such as rodents) might be infected with the virus as they consume human waste that is laden with the virus, thus causing new infected wild animal populations to establish themselves in traditionally non-endemic areas (Murphy and Ly, 2022; Peiro-Mestres et al., 2022).

Researchers are investigating whether monkeypox can be spread when a person does not have symptoms, or if people with monkeypox symptoms are more likely to spread the virus, and also to determine if the virus is spread more easily through feces, semen, vaginal fluid, or urine of symptomatic patient. To date, the disease seems to be having difficulties spreading effectively, however, the possibility that it can be transmitted through intimate or sexual contact ought to alarm infectious disease specialists in conducting further research to determine how the disease spreads so it can be prevented from spreading worldwide (Zambrano et al., 2022).

## 8 Clinical manifestation

Most monkeypox cases are self-limiting, with clinical manifestation as detailed in **Figure 4**. In children, complications are more likely to occur if they have been exposed to a high level of virus exposure, they have a poor health status, and they have complications of a severe nature. There is a possibility that underlying immune deficiencies will result in worse outcomes. In spite of vaccination against smallpox being protective in the past, now that campaigns of smallpox vaccination have stopped since the disease was eradicated globally, young people may be more susceptible to monkeypox. In monkeypox, lymphadenopathy distinguishes it from other similar diseases (chickenpox, measles, smallpox) and the skin eruption usually appears within 1-3 days of the fever onset. The rash can manifest as a pimple or blister and may be itchy or painful. Some patients may experience all symptoms at once, whereas others may experience only a few. Also, it affects the mucous membranes of the oral cavity, the genitalia and the conjunctiva. The disease begins with macules (flat lesions) then progresses to papules (slightly raised firm lesions), pustules (fluid filled yellow lesions), vesicles (fluid-filled clear lesions) and crusts that fall off over time. In some cases, there can be few to several thousand lesions. Severe cases can result in large areas of skin sloughing off caused by the coalescence of lesions (MacNeil et al., 2009; Adler et al., 2022; European Medicines Agency, 2022; Gilbourne et al., 2022; Monkeypox: What to know, 2022).

Asymptomatic infection may occur in some cases, but it is not known to what extent. In the general population, monkeypox has historically had a case fatality rate between 0 and 11%, with a higher rate among younger children. Recent years have seen a 3–6% case fatality rate (Ministry of Health and Family Welfare, 2020).

As a result of monkeypox associated encephalitis, patients often experience pharyngitis, fever, anorexia, headache, weakness, adenopathy, and a vesiculo-papular rash. A very slow electroencephalogram and diffuse oedema found on cortical magnetic resonance imaging were associated with amplification of meninges in the thalamus and partial cortex as well as signal abnormalities. However, within 5-6 days polymorphonuclear pleocytosis in cerebrospinal fluid (CSF) may decrease and primarily consist of lymphocytes (Shafaati and Zandi, 2022).

## 9 Differential diagnosis

When considering the differential diagnosis, other rash illnesses should also be considered, such as measles, chickenpox, scabies, bacterial infections, and medications associated allergies. When monkeypox is in its prodromal stage, lymphadenopathy distinguishes it from chickenpox or smallpox (World Health Organization, 2022).

A monkeypox sample must be collected by health workers and transported safely to a laboratory that has the necessary capabilities if monkeypox is suspected. A laboratory test and the type of specimen determine whether monkeypox is confirmed or not. It is therefore imperative that specimens are packaged and shipped must comply with international and national regulations. In terms of accuracy and sensitivity, polymerase chain reaction (PCR) is the preferred laboratory test. Ideally, monkeypox diagnostic samples should come from skin lesions such as vesicles, pustules, and crusts. Biopsies can be performed where feasible. Keeping lesions cold and storing them in dry and sterile tubes (no viral transport media) is essential. But, due to the short duration of viremia, blood tests for PCR are usually inconclusive and should not be routinely conducted in patients of monkeypox after symptoms begin (Ministry of Health and Family Welfare, 2020).

Also, serological cross-reactivity between orthopoxviruses makes antibody and antigen detection method ineffective from providing monkeypox specific confirmation. Hence it is not recommended to use serology or antigen detection methods for case investigation where resources are limited. Furthermore, a false positive result may also result from recent vaccination with an orthopoxvirus vaccine (e.g. those vaccinated before smallpox eradication or more recently due to a higher risk). So, for interpretation of test results, patient symptoms information must be included with specimens (Reed et al., 2004; Reynolds et al., 2006; Dubois and Slifka, 2008). It may be helpful to detect orthopoxvirus induced encephalitis. A CSF IgM reaction against orthopoxvirus may help detect encephalitis caused by orthopoxvirus (Sejvar et al., 2004).

## 10 Prevention strategies

**Figure 5:**
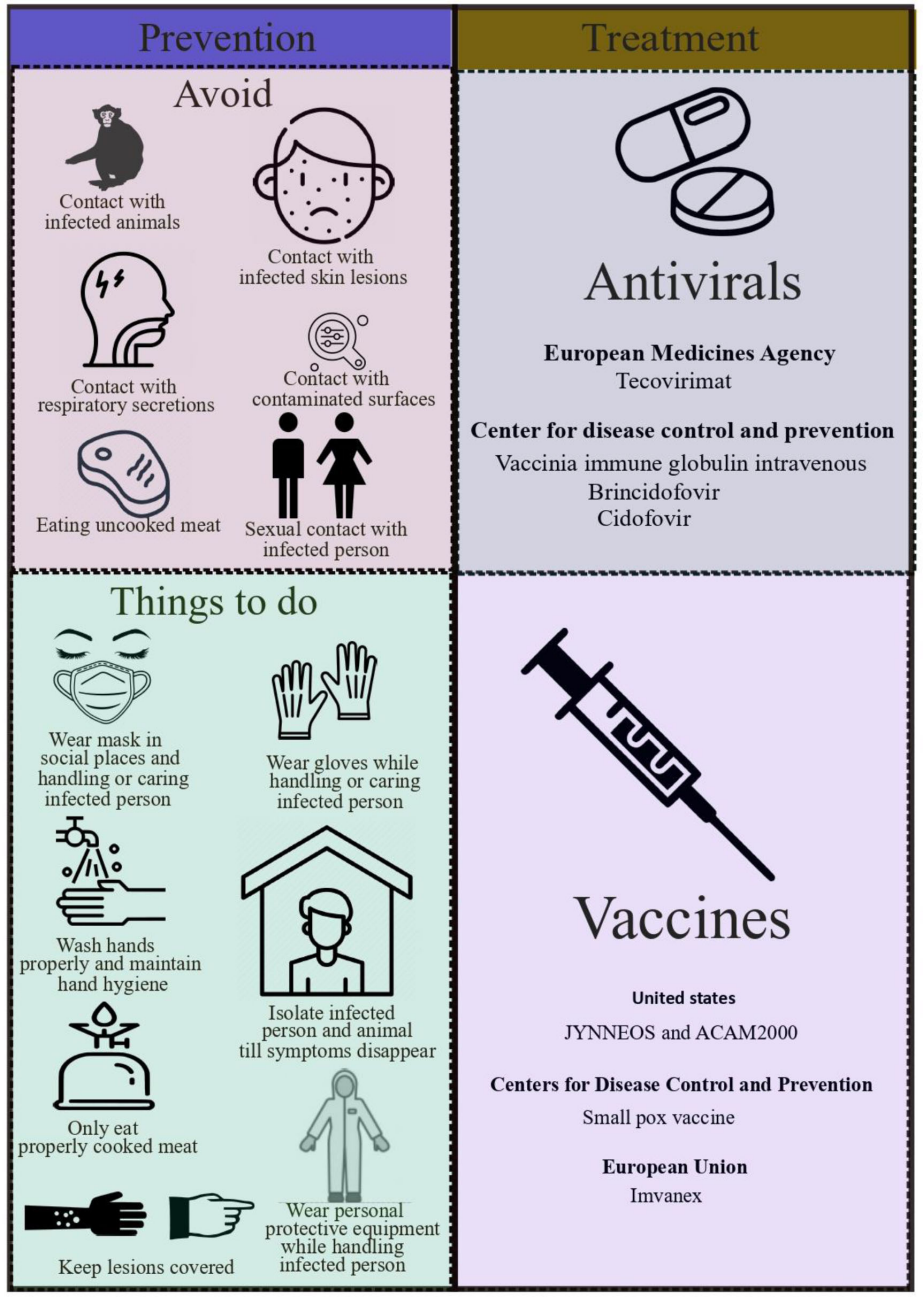
Representing prevention and treatment of monkeypox.

Educating people about risk factors and ways to reduce exposure is the primary strategy for preventing monkeypox (**Figure 5**). Research is currently being conducted to determine whether vaccination is an appropriate and feasible preventative or control measure against monkeypox. To protect the public’s health, it is important to investigate all possible modes of transmission since the source of this outbreak is still being investigated. Many countries have or developing policies that offer vaccines to health workers, laboratory personnel, and rapid response teams who may be at risk (Health ministry issues guidelines for monkeypox management - the statesman, 2022).

Also to reduce the risk of transmission between humans, surveillance and rapid detection of new cases are crucial for outbreak containment. Standard infection control precautions should be followed by health workers caring for patients or handling specimens from those patients with monkeypox virus infection. The patient should be cared for by persons previously vaccinated against smallpox if possible. Make sure you are using appropriate personal protective equipment (PPE) when caring for those with symptoms, including gloves, a mask, and a gown. Personnel who are trained and equipped with the proper equipment should handle samples suspected of being infected with monkeypox virus. According to WHO guidelines for transporting infectious substances, patient specimens must be packaged triple to ensure their safety (Health ministry issues guidelines for monkeypox management - the statesman, 2022). Keep your sexual partner/s informed about any recent illness, including sores or rashes, and avoid close contact with anyone who has symptoms such as sores or rashes. To reduce the risk of a resurgence of infection, a 21-day self-isolation period and other preventive measures have been implemented UK Health Security Agency, 2022.

Furthermore to prevent monkeypox, some countries have enacted regulations restricting rodent and non-human primate importation. Monkeypox infected captive animals or that might have come into contact with them should be quarantined immediately, handle with standard precautions, and observed for 30 days to monitor monkeypox symptoms (Health ministry issues guidelines for monkeypox management - the statesman, 2022).

## 11 Therapeutics

A specific treatment does not exist for monkeypox viral infections. Monkeypox and smallpox viruses have genetic similarities, so smallpox related antiviral drugs and vaccines may be effective against monkeypox. It is essential that patients receive fluids and food in order to maintain a healthy nutritional status and infections caused by secondary bacteria should be treated accordingly (Health ministry issues guidelines for monkeypox management - the statesman, 2022).

Tecovirimat, an antiviral agent previously approved by the United States Food and Drug Administration (FDA) for smallpox, has been now approved by the European Medicines Agency (EMA) for treatment of monkeypox after animal and human studies. Tecovirimat (previously ST-246), a small molecule that inhibits viral egress, has been demonstrated to be effective against vaccinia, cowpox, camelpox, ectromelia (mousepox), and variola viruses (Yang et al., 2005). Through screening of more than 350,000 compounds, tecovirimat was discovered to target a gene that produces p37, an envelope protein essential for extracellular virus production (Bolken and Hruby, 2010). In mice, tecovirimat was proven to be effective and safe when administered orally twice daily at 50 mg/kg of body weight for 14 days before or shortly after infection. When used at a concentration (EC50) of ≤0.07 μM, it suppresses viral multiplication by 50% in *in vitro* (Quenelle et al., 2007). In a ground squirrel model of disease, tecovirimat was effective in saving all animals that were given the drug on days 0, 1, 2, and 3; in contrast, all animals in the placebo group died (Nalca et al., 2008). It was clear from these findings that further human studies were necessary. In patients with weakened immune systems, antivirals such as tecovirimat may be helpful in preventing severe illness. If tecovirimat will be used for patient care, WHO recommends monitoring it in a clinical research setting and have prospective data collection (Centers for Disease Control and Prevention, 2022; World Health Organization, 2022). Recently, Phase 1 and Phase 2 clinical trials involving an oral formulation of it was funded by National Institute of Allergy and Infectious Disease (NIAID and Biomedical Advanced Research and Development Authority (BARDA), part of the USA Department of Health and Human Services (NIH: National Institute of Allergy and Infectious Diseases, 2022).

Monkeypox can be prevented through vaccination if administered before or soon after exposure. There are currently two monkeypox vaccines available in the USA through the Strategic National Stockpile: JYNNEOS and ACAM2000. For adults 18 years and older, JYNNEOS is licensed. At least four weeks apart, two doses have to be administered in the upper arm. Itching, redness and swelling are the most common reactions people have at the injection site after receiving the JYNNEOS vaccine. Public health has prioritized the JYNNEOS vaccine for the following groups: known close contacts of monkeypox cases identified by case investigations, tracing of contact, and assessments of risk exposure, and for individuals who attended an event where monkeypox was present. Advisory committee on immunization practices(ACIP) also recommends monkeypox vaccinations for laboratory and clinical workers who conduct monkeypox testing and collect monkeypox specimens (Centers for Disease Control and Prevention, 2022). Moreover, European Union (EU) has also approved Imvanex as a vaccine against monkeypox disease as of 22 July 2022. Later, Imvanex has been authorized for the treatment of smallpox and vaccinia virus in the EU (European Medicines Agency, 2022).

**Figure 6:**
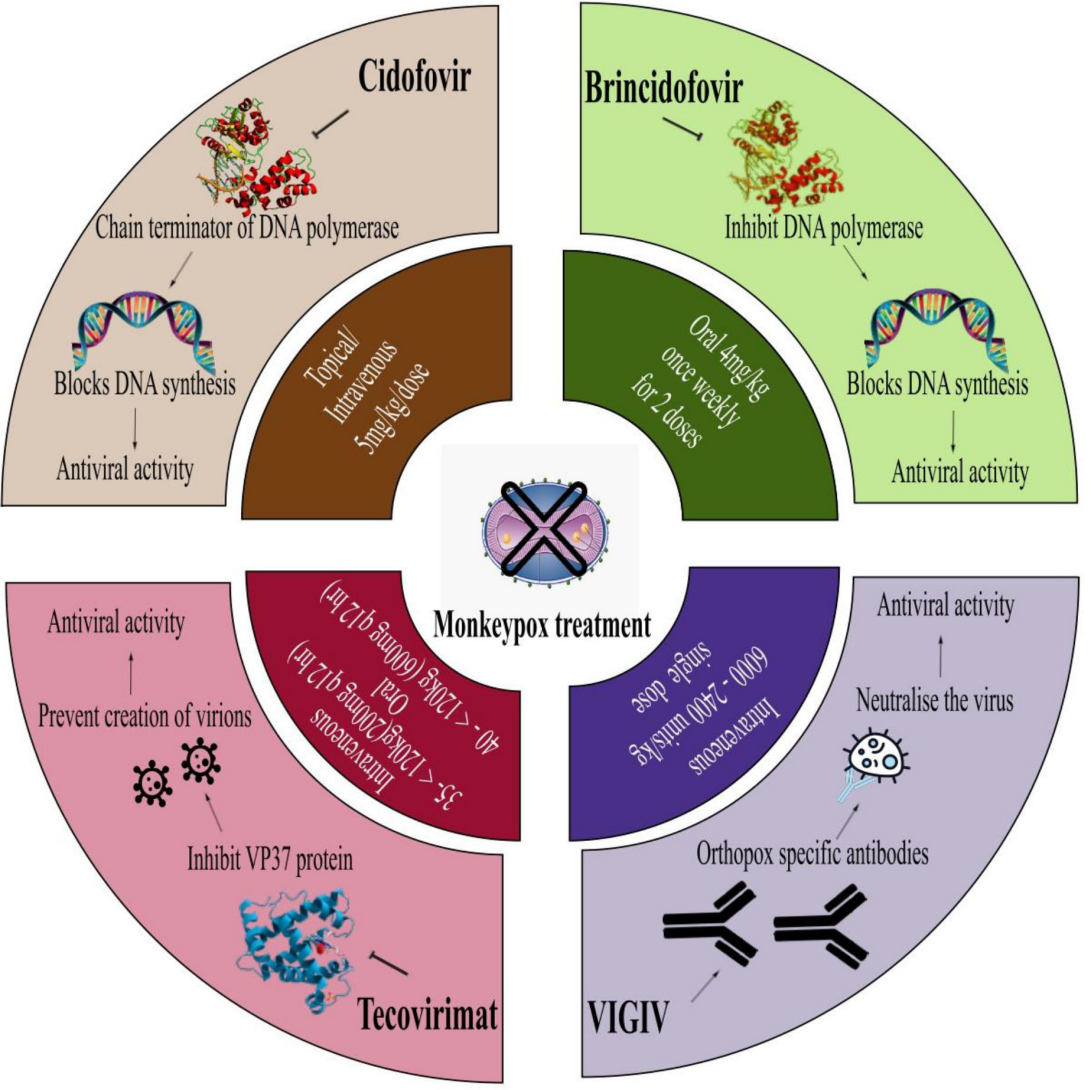
Representing mechanism and dose of various monkeypox treatments.

Furthermore, the Center for Disease Control and Prevention (CDC) recommends administration of vaccinia immune globulin intravenous(VIGIV), cidofovir and Bricidofovir to treat monkeypox. Earlier, VIGIV was used for treating complications caused by vaccinating against vaccinia, such as generalized vaccinia severe, eczema vaccinatum, vaccinia infections among skin diseases, progressive vaccinia and aberrant infections caused by vaccinia virus (except in isolated cases of keratitis). And as an antiviral, FDA has approved cidofovir to treat cytomegalovirus (CMV) retinitis among AIDS patients and Bricidofovir for treating smallpox in adults and children, including neonates since June, 4, 2021. There is a possibility that Bricidofovir has a better safety profile than cidofovir. There have been no serious renal toxic effects or other adverse reactions associated with Bricidofovir treatment for cytomegalovirus infection (**Figure 5**). Dose and possible mechanism of these vaccines are represented in **Figure 6** (Centers for Disease Control and Prevention, 2022).

Despite their effectiveness in animal studies against orthopoxviruses, no data indicate that they are effective in humans infected with monkeypox virus. Patients with severe T-cell deficiencies and who are contraindicated from smallpox vaccination after monkeypox exposure should take VIGIV. Cidofovir or brincidofovir should be given to treat monkeypox outbreaks in patients who have severe immunodeficiency (Kugelman et al., 2014; Centers for Disease Control and Prevention, 2022). All these vaccines suggested to be used in monkeypox should be further clinically proved.

## 12 *In silico* findings: Possible drug targets

In silico studies have shown that the genome of monkeypox are identical to the central regions that encode essential enzymes and proteins to the genome of smallpox. Despite the fact that monkeypox genome nomenclature resembles universal nomenclature, certain regions differ encoding for virulence. There is a great deal of research being done on the vaccinia virus, as it is used in vaccinations against other types of poxviruses and has lower risk than smallpox. Each of the three agents of monkeypox, smallpox, and vaccinia has nearly 197, 186, and 190kb of genome size respectively. The genomes of all poxviruses are linear and have double-stranded DNAs. Since other poxvirus homologues have similar characteristics and naming problems, the authors of that study refer to their targets by the universal nomenclature gene name based on the vaccinia nomenclature. A50R, A48R, D13L protein trimer complex, F13L, and E are five poxvirus targets examined in that study. A number of studies and reviews have proposed that I7L be used as a useful target for intervention. Based on these targets, an in silico study was conducted on the active residues of monkeypox and eight potential repurposable drugs were identified. In this list of eight drugs, they find nilotinib for A50R, rutaecarpine and NMCT for A48R, naldemedine and hypericin for F13L, simeprevir for D13L, and lixivaptan and fosdagrocorat for I7L. As the drugs show promise in an in silico model, they should also be tested in clinical trials to see if they can combat disease effectively (Prazsák et al., 2018; Senkevich et al., 2021; Akash et al., 2023; Varghese et al., 2023b).

## 13 Case studies and therapeutic intervention

Few case studies cases pertaining to this outbreak of monkeypox available online are discussed below in **Table 2** to understand the presentation and treatment provided. It is crucial for health authorities and clinicians to take into account the diagnosis of monkeypox in all patients, as cases in which patients have other sexually transmitted infections make it difficult to diagnose. For this reason, clinicians should always test suspected cases for monkeypox as part of their differential diagnosis. Further, it was noticed that antibiotics, antivirals and analgesics are given to these patients and recovery is shown. Although, this data is insufficient to propose any valid treatment, but these leads can be taken in consideration and validate in more number of patients. Also there are 8 studies yet found to be registered at clinicaltrial.gov that are related to monkeypox. The studies are compiled in **Table 3**. Among them are three interventional studies (IMVAMUNE^®^, ST-246, MVA vaccine (IMVANEX^®^ and JYNNEOS^®^)), four observational studies, and one study to expand access to tecovirimat. Recruitment of patients are still ongoing for the studies (Search of: Monkeypox - list results - ClinicalTrials.gov, 2022). In future, the findings of these studies will hopefully provide some scientific results.

**Table 2:** Overview of the clinical course and outcome of cases from recent outbreak of monkeypox.

Country	Age (years)	Gender	Comorbidity	Treatment	Clinical presentation	Infection site	Outcome	Hospitalization require (days)	References
Romania	26	Male	HIV	-*	Vesicular and pustular rash, few lesions, hyperemia of pharynx, pseudo-membranous appearance, thrush, enlarged lymph nodes	Rash & lession- Buttocks, neck, trunk, sole, upper and lower limbs; Lymph nodes- cervical and inguinal region	-*	-*	(Oprea et al., 2022)
Italy	34	Male	HIV	Cephalosporins	Painless ulceration after sex with men, high fever, chills, painful enlargement, papulovesicles, itchy rashes,	Lymph node- left inguinal; Rashes- forehead, perianal, left tonsil	-*	-*	(Bížová et al., 2022)
Italy	33	Male	HIV	-*	Asthenia, malaise, anorexia, papular lesion, ulceration, respiratory symptoms, fever	Elbow, perianal	-*	-*	(Bížová et al., 2022)
Italy	26	Male	-*	Amoxicillin potassium cluvunate 3 g/d for 8 days and cidofovir 5 mg/kg day 1 and 7	Chills, sweats, lesion followed by high fever, lymphadenopathy	Nose, limb	Recovery	Yes(8)	(Moschese et al., 2022)
Italy	35	Male	-*	Analgesic therapy	Vesicular rash followed by fever, lymphadenopathy	Head, trunk, limbs	Recovery	Yes (5)	(Moschese et al., 2022)
Italy	34	Male	HIV	None	Lesion followed by fever, lymphadenopathy	Perianal, face, foot, arm	Recovery	Yes(8)	(Moschese et al., 2022)
Italy	37	Male	HIV	Ceftriaxone 2 g/d for 7 days and daptomycin 500 mg/d for 5 days	Skin lesion followed by fever, headache, lymphadenopathy	Inguinal, scrotum, penis, face	Recovery	Yes(13)	(Moschese et al., 2022)
France	-*	Male	-*	No specific treatment	Fever, intense fatigue, chills, myalgia, several anal pain, lymphadenopathy and sore throat	-*	Recovery	No	(Vallée et al., 2022)
Taiwan	20	Male	-*	-*	Fever, muscle pain, sore throat, skin rash and lymph node swelling in the groin	-*	Recovery	No	(Yang et al., 2022)

**Table 3:** Representing data of case studies on monkeypox registered with clinicaltrial.gov.

NCT05476744	Viral clearance and epidemiological characteristics in patients with monkeypox	Observational	Cohort, prospective	Spain	No	100	Recruiting	Viral clearance in skin lesions, blood and oropharyngeal swabs
NCT05443867	Monkeypox a symptomatic shedding: evaluation by self-sampling MPX-ASSESS	Observational	Cohort, prospective	Belgium	No	140	Recruiting	Secondary attack rate of monkeypox virus infection in contacts, defined by PCR positivity on any sample, rate of seroconversion in contacts, defined as a positive IgG (immunoglobulineG) for monkeypox and proportion of seroconversion in PCR positive contacts vs PCR negative.
NCT05438953	Follow-up of contact at risk of monkeypox infection: a prospective cohort study	Interventional	Non-randomized, parallel assignment, open label, prevention	France	MVA vaccine (IMVANEX^®^ and JYNNEOS^®^)	226	Recruiting	Proportion of failure of MVA vaccine, assess early vaccine humoral immunogenicity and early vaccine humoral immunogenicity after one, two doses
NCT02977715	IMVAMUNE^®^ smallpox vaccine in adult healthcare personnel at risk for monkeypox in the DRC	Interventional	Single group, open label, prevention	DRC	IMVAMUNE^®^	1600	Active, not recruiting	Proportion of participants who develop suspected or confirmed monkeypox virus infection following receipt of IMVAMUNE and proportion of participants who have Orthopoxvirus antibody responses on days 0, 14, 28, 42, 180, 365, 545, and 730 days after the receipt of the first dose of vaccine
NCT05058898	A one health study of monkeypox human infection	Observational	Case-control, prospective	Central African Republic	Blood samples, scabs and pus samples	280	Recruiting	Proportion of monkeypox cases occurring following interhuman exposures, zoonotic exposures, measurement of the effective reproduction rate R in CAR according to the level of immunity (smallpox vaccine immunity or Orthopoxvirus-related post disease immunity)
NCT03745131	Cohort study of healthcare workers receiving Imvanex^®^	Observational	Cohort, prospective	United kingdom	Blood draw	120	Completed	Antibody responses to first dose of Imvanex^®^, antibody titres following first dose of Imvanex^®^ and antibody responses to second dose of Imvanex^®^
NCT02080767	Tecovirimat (ST-246) treatment for orthopox virus exposure	Expanded access	–	–	Tecovirimat	–	Available	–
NCT00728689	Phase I trial of an investigational small pox medication	Interventional	Randomized, crossover, triple masking, treatment	United states	ST-246 Days 1 - 3	12	Completed and has results	Pharmacokinetic Parameters for a Single Dose of ST-246 form I vs. form V: t½, form I vs. form V: AUC0-τ and form I vs. form V: AUC0-∞
**NCT number**	**Study title**	**Study type**	**Study design**	**Country**	**Interventions**	**Number enrolled**	**Status**	**Outcome measures**

## 14 Possible plant-based strategies and probable mechanism involved to manage upcoming monkeypox cases

**Figure 7:**
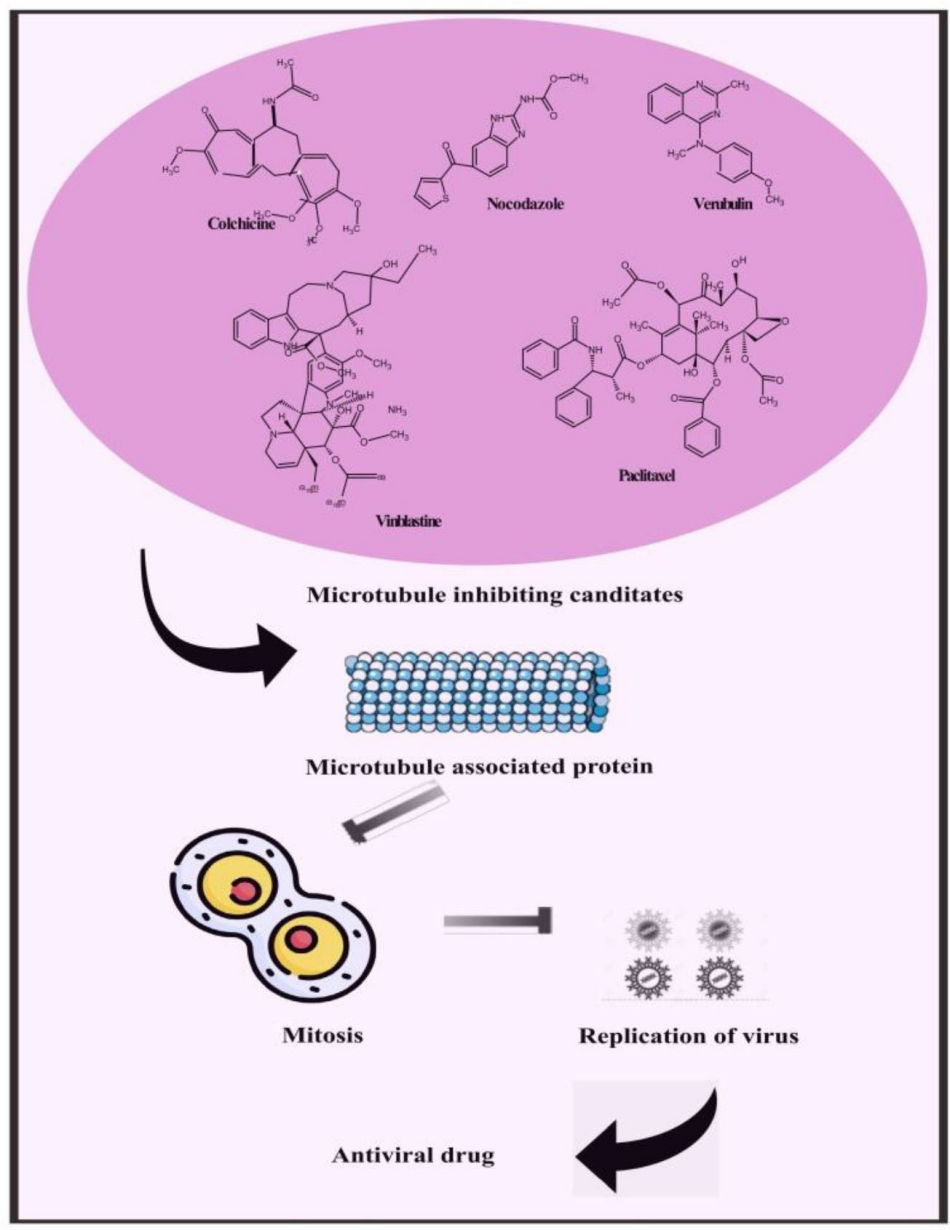
Representing possible leads constituents to mitigate monkeypox by microtubule inhibiting candidate.

Besides providing scaffolding for cells and facilitating cellular long-distance traffic, microtubules serve as important components of multiple biological processes. It is astounding that viruses are often able to move actively into cells by using the cytoskeleton transportation machinery. As part of their replication cycle, viruses often interact with the cytoskeleton and require an intact microtubule network. Microtubule-dependent motors are involved in transporting intact virions and capsids to replication sites and exiting replication sites to the plasma membrane for some viruses. Upon maturation of the endosomes, some viruses move along microtubules with a characteristic vesicular motion that targets either kinesins or dyneins. Pharmacological modulation of microtubules has been hypothesized to interfere with virus replication and spread, demonstrating their potential as broad-spectrum antivirals. In contrast, an pharmacological interventions against viral infections must be tightly controlled in order to prevent the compromise of physiological functions of cells. Therefore, we suggest microtubule targeting agents to inhibit viral replication in monkeypox (**Figure 7**) (Vale, 1987; Gundersen and Cook, 1999; Ridley et al., 2003; Khodjakov and Rieder, 2009; Mogilner and Keren, 2009; van der Vaart et al., 2009; Stehbens and Wittmann, 2012; Zulkipli et al., 2015).

Compounds targeted at microtubules from natural sources and synthetic compounds such as Colchicine, Nocodazole, Vinblastine, Paclitaxel, Vincristine, Podophyllotoxin, Combretastatins, Noscapine, Vindesine, Vinorelbine, Vinflunine, Docetaxel, Cabazitaxel, Larotaxel, Tesetaxel, Ombrabulin, Fosbretabulin, Crolibulin and Verubulin are potent antiviral leads that can also be an effective treatment for monkeypox (Kaul et al., 2019).

Also, the traditional use of several plant species and their extracts investigated for their antiviral properties has been documented. An array of plant extracts including *Pterocaulon sphacelatum, Dianella longifolia* var. *grandis, Euphorbia australis, Scaevola spinescens, Pittosporum phylliraeoides* var. Microcarpa*, Azadirachta indica, Eremophila latrobei* subsp. Glabra*, Opuntia streptacantha, Nerium indicum, Bergenia ligulata* and *Holoptelia integrifolia* exhibited antiviral effects towards a variety of DNA viruses in experimental models. The fact that monkeypox is a DNA virus makes it a viable candidate for inhibiting actions of these herbs (Semple et al., 1998; Abubakar et al., 2022). Furthermore, a study suggests that *Acacia nilotica* (L.) Delile, *Adansonia digitata* L., *Aframomum melegueta* K. Schum., *Allium sativum* L., *Alstonia boonei* De Wild, *Anogeissus leiocarpus* (DC.) Guill. & Perr., *Azadirachta indica* A. Juss., *Balanites aegyptiaca* (L.) Delile, *Calotropis procera* (Aiton) Dryand, *Carica papaya*, *Cissus populnea* Guill. & Perr., *Citrullus lanatus* (Thunb.) Matsum. & Nakai, *Combretum micranthum* G. Don., *Detarium senegalense* J.F. Gmel., *Diospyros mespiliformis* Hochst. ex A.DC., *Eleusine coracana* (L.) Gaertn., *Euphorbia hirta* L, *Ficus platyphylla* Delile, *Ficus polita* Vahl, *Guiera senegalensis* J.F. Gmel., *Lagenaria breviflora* (Benth.) Roberty, *Lawsonia inermis* L, *Mangifera indica*, *Maytenus senegalensis* (Lam.) Exell, *Momordica charantia* L., *Moringa oleifera* Lam., *Nigella sativa* L., *Olea europea* L, *Parinari macrophylla* Sabine, *Piper guineense* Schumach. & Thonn., *Sterculia setigera* Delile, *Tamarindus indica*, *Terminalia avicenoides* Guill. & Perr., *Vernonia amygdalina*, *Vitellaria paradoxa* C.F. Gaertn, *Viscum album* L. and *Ziziphus mauritiana* Lam. were among the most commonly used plants for treating monkey pox in different country regions. As of now, monkeypox does not have a well-established and proven treatment. It is therefore essential to evaluate the molecular mechanism underlying these plant-based treatments and to verify their pharmacological claims in different experimental models as to their ability to mitigate monkeypox virus (Jassim and Naji, 2003).

## 15 Conclusion and future perspectives

Each day, a growing number of monkeypox cases are reported, and more cases are expected in the near future. Most cases have been found among men, of whom several are gay or bisexual. A rash was the most common symptom that drove patients to seek medical attention. For the first time, a chain of transmission has been reported in Europe without any epidemiological connection to West or Central Africa. As compared to the Central African strain (ZAI-96), the nucleotide sequences of the three West African strains (SL-V70, COP-58, and WRAIR-61) differ by only 0.55-0.56%. BR-203, BR-209, and COP-C3L orthologs may play a role in the virulence differences between the strains. Multiple molecular mechanisms are stated in this study which explain how monkeypox viruses produce symptoms such as apoptosis by NOXA and caspase-3 upregulation, Bcl-2, PUMA, and PAK2 downregulation, targeting mitochondria, expression of histone, modification of histone posttranslational, dynamic exchanges of chromatin, packaging of DNA, ROCK1 and actin components regulating cytoskeletal dynamics. In order to reduce the risk of future outbreaks, it is imperative to act quickly and stop community transmission. Several key features need to be acknowledged and addressed if we hope to prevent monkeypox and minimize unintentional harms. Additionally, it is likely that an early cluster is responsible for the most cases among men who have sexual relationship with men in the current outbreak It is important to identify monkeypox cases early so that officials of public health can identify potential contacts, their vulnerability, isolate them appropriately, keep track of symptoms, and possibly administer vaccinations. Whenever dermatologists are able to evaluate a patient with papulo-vesiculo-pustular or vesiculo-pustular lesions, should be evaluated for presence of monkeypox. If a diagnosis is suspected or confirmed, public health systems should be contacted quickly. Public health authorities should therefore support those exposed to monkeypox or quarantined monkeypox patients, especially because the pathogen has a prolonged incubation period and is infectious for a long period of time. Studies have revealed the possible plants-based leads to mitigate the monkeypox. These leads should attract the attention and more studies to validate these treatments to help mitigating monkeypox pandemic.

## Author contributions

RuS, writing – original draft and formal analysis. K-TC, editing. RoS, conceptualization, editing and supervision. All authors contributed to the article and approved the submitted version.
